# Curcumin and long-chain Omega-3 polyunsaturated fatty acids for Prevention of type 2 Diabetes (COP-D): study protocol for a randomised controlled trial

**DOI:** 10.1186/s13063-016-1702-9

**Published:** 2016-11-29

**Authors:** Rohith N. Thota, Shamasunder H. Acharya, Kylie A. Abbott, Manohar L. Garg

**Affiliations:** 1 Nutraceuticals Research Group, School of Biomedical Sciences and Pharmacy, University of Newcastle, 305C Medical Sciences Building, Callaghan, NSW 2308 Australia; 2 Department of Endocrinology, John Hunter Hospital, Hunter New England Local Health District, New Lambton Heights, NSW Australia

**Keywords:** Curcumin, Docosahexaenoic acid, Eicosapentaenoic acid, Omega-3 fatty acids, Inflammation, Insulin sensitivity, Prediabetes and type 2 diabetes

## Abstract

**Background:**

Lifestyle interventions, including increase in physical activity and dietary counselling, have shown the ability to prevent type 2 diabetes (T2D) in high-risk state individuals, but the prevalence is still skyrocketing in Australia, in line with global prevalence. Currently, no medicines are approved by the Therapeutic Goods Administration in Australia for the management of prediabetes. Therefore, there is a need of developing a safer, biologically efficacious and cost-effective alternative for delaying the transition of individual health state from prediabetes into T2D. In the current trial we propose to evaluate the effects of curcumin and/or long-chain omega-3 polyunsaturated fatty acids on improving glycosylated haemoglobin as a primary outcome, along with secondary outcomes of glycaemic indices, lipid profile and inflammatory parameters.

**Methods/design:**

Eighty individuals diagnosed with prediabetes, aged between 30 and 70 years, will be randomly assigned to double placebo, curcumin alone, fish oil alone or double active groups according to a computer-generated randomisation sequence for 12 weeks. At baseline and post-intervention visits participants will be asked to provide blood samples and undergo body composition measurements. A blood sample is used for estimating glycaemic profiles, lipid profiles and inflammatory parameters (C-reactive protein, whole blood cell count, adiponectin, leptin, interleukin-6). The interim visit includes review on compliance with supplements based on capsule log and capsule count, adverse events and anthropometric measurements. In addition to these procedures, participants provide self-reported questionnaires on dietary intake (using a 3-day food record), a physical activity questionnaire and medical history.

**Discussion:**

This trial aims to determine whether curcumin and/or long-chain omega-3 polyunsaturated fatty acids affect surrogate markers of glycaemic control which is relevant to delaying T2D. To date 38 participants completed the trial. No changes have been made to the clinical protocol post recruitment. If successful, this trial will provide considerable evidence for performing a larger trial to investigate whether this combination can be administered for preventing or delaying the onset of T2D in high-risk individuals.

**Trial registration:**

ACTRN12615000559516, registered on 29 May 2015).

**Electronic supplementary material:**

The online version of this article (doi:10.1186/s13063-016-1702-9) contains supplementary material, which is available to authorized users.

## Background

Type 2 diabetes (T2D) is a chronic and noncommunicable metabolic disorder characterised by hyperglycaemia resulting from defective secretion and/or action of insulin. The prevalence of T2D has been increasing rapidly worldwide at an alarming rate due to over nutrition, physical inactivity, urbanisation (especially in developing countries) and genetic predisposition [1–4]. People with prediabetes, who are at a high risk of developing T2D, have intermediate glucose levels that are elevated but not high enough to be diagnosed as T2D. They are classed as having either impaired glucose tolerance (IGT), impaired fasting glucose (IFG) or both. In 2015, over 318 million people were living with prediabetes worldwide, and this number is expected to rise to 471 million by 2035 [5]. Five to ten percent of the individuals diagnosed with prediabetes continue to progress to overt T2D annually [6]. Despite its complex and heterogeneous nature, T2D often coexists with obesity, hypertension and dyslipidaemia which are also common features of ageing [7–10]. Recent advances in molecular biology have provided in-depth understanding of the mechanisms that are involved in the pathobiology of T2D highlighting the role of glucotoxicity, lipotoxicity, oxidative stress and endoplasmic reticulum stress [11–13]. An elevated level of subclinical inflammation for prolonged periods is believed to be the primary trigger preceding all of the above mechanisms [14]. These pathological mechanisms mediate a reduction in insulin-stimulated glucose uptake leading to insulin resistance. Insulin resistance, present throughout the prediabetic stage, acts as a conduit between the progression of the subclinical inflammatory state to T2D.

The presence of insulin resistance in prediabetes also modulates the risk of developing T2D, and individuals with combined IGT and IFG are at greater risk of developing T2D than those with IFG or IGT alone [6, 15]. Insulin resistance is prevalent in both the cases, but people with IFG have more hepatic insulin resistance and those with IGT have more skeletal muscle resistance with less effect on hepatic insulin sensitivity [16, 17]. Along with insulin resistance, beta-cell dysfunction is present in both IFG and IGT individuals. However, they differ in time of insulin secretion response during an oral glucose tolerance test (OGTT). Individuals with IGT have both impaired early and late insulin response, in contrast to IFG individuals where only early phase insulin response is impaired [16]. These observations indicate that insulin resistance and moderate beta-cell dysfunction is already present in individuals with IFG and IGT (prediabetes).

Diabetes prevention studies have found that lifestyle interventions aiming at weight reduction through dietary intake changes and increase in physical activity significantly reduce the risk of developing T2D [15, 18]. Lifestyle interventions resulting in weight loss also improved insulin sensitivity and beta-cell function [19–21]. However, maintaining weight loss and physical activity is very difficult over longer periods of time. Lack of compliance with these interventions is a barrier for effectiveness in halting the progression to T2D in high-risk individuals, indicating that dietary and lifestyle modifications alone are insufficient. As prediabetes is not a full-blown disease state, to date, there are no approved drugs for its management. Lack of compliance with lifestyle interventions and pharmacological agents highlights the necessity of safe and efficacious interventions for the management of prediabetes.

Subclinical inflammation, being the primary trigger for many pathological mechanisms in the development of T2D, anti-inflammatory bioactives, such as curcumin and long-chain omega-3 polyunsaturated fatty acids (LCn-3PUFA), appear to be an attractive strategy to delay the risk of developing T2D in individuals with prediabetes. These bioactive compounds influence multiple numbers of physiological mechanisms and have relatively higher safety profiles [22, 23]. These bioactives could provide beneficial effects in insulin resistance and T2D by the following pathways: (1) down-regulating inhibitor of kappa B kinase (IKK)/nuclear factor kappa B (NFκB), and c-Jun N-terminal kinase (JNK) pathways for lowering chronic low-grade inflammation, (2) scavenging reactive oxygen species (ROS) and reducing oxidative stress, (3) providing cytoprotection for improving beta-cell function, (4) reducing the accumulation of fatty acid metabolites for increasing insulin sensitivity, (5) up-regulation of 5' AMP-activated protein kinase (AMPK), and (6) modulating peroxisome proliferator-activated receptor gamma (PPARγ) agonistic activity.

Some preclinical studies have reported synergistic anti-inflammatory effects of curcumin and docosahexaenoic acid (DHA) combination. These observations suggest that curcumin might provide synergistic anti-inflammatory effects with LCn-3PUFA in the prevention of T2D [24–26]. In this study we aim to perform a 2 × 2 factorial, double-blinded, randomised placebo-controlled trial to evaluate the complimentary or synergistic effects of curcumin and/or LCn-3PUFA primarily on glycaemic profiles and in individuals with prediabetes (IFG, IGT or both). In addition to these parameters we are also measuring lipid profile and inflammatory parameters to determine the effects of curcumin and/or LCn-3PUFA on cardiovascular risk factors and chronic low-grade inflammation.

## Method/design

**Fig. 1:**
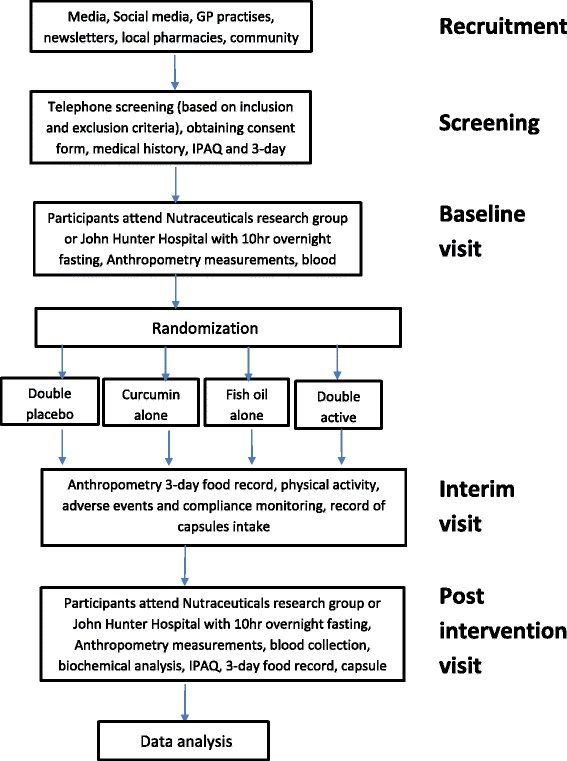
Trial protocol flow chart

This is a 12-week 2 × 2 factorial, double-blinded (study investigator and participants), randomised placebo-controlled intervention trial for preventing or delaying the onset of T2D using curcumin and/or fish oil as supplements. Eighty individuals diagnosed with prediabetes (IFG fasting glucose 6.1–6.9 mmol/L; IGT-2 h OGTT plasma glucose ≥7.8 mmol/L and ≤11.1; glycosylated haemoglobin (HbA1c) 5.7–6.5 mmol/L) aged between 30 and 70 years will be screened for enrolment at two sites; Nutraceuticals Research Group (NRG) and John Hunter Hospital (JHH) in Newcastle, NSW, Australia. Potential participants who meet the inclusion and exclusion criteria will be randomised to receive double placebo (corn oil capsules or curcumin placebo tablets), curcumin plus placebo matching for fish oil, fish oil plus placebo matching for curcumin or both curcumin and fish oil. Participants visit the trial sites three times at baseline (0 weeks), interim (6 weeks) and post intervention (12 weeks). Figure 1 shows a flow chart of the study design and the Standard Protocol Items: Recommendations for Interventional Trials (SPIRIT) Checklist is presented as Additional file 1.

### Study aims

The primary aim is to evaluate the potential of curcumin and/or LCn-3PUFA for improvements in the HbA1c in individuals with prediabetes. Secondary outcomes include: fasting plasma glucose, insulin, fructosamine, insulin sensitivity (Homeostatic Model Assessment), lipid profile (total cholesterol (TC), triglycerides (TG), high-density lipoprotein cholesterol (HDL-C), low-density lipoprotein cholesterol (LDL-C)), C-reactive protein (CRP), interleukin (IL)-1β, IL-6, adiponectin and leptin. Erythrocyte fatty acid composition and plasma curcumin content are measured to determine compliance with the intervention.

### Inclusion criteria

To be eligible to participate in the trial, participants should be within the age range of 30–70 years. Their Body Mass Index (BMI) must lie between 25 and 45 kg/m^2^. They must be diagnosed either with IFG (fasting glucose 6.1–6.9 mmol/L), IGT (2-h OGTT plasma glucose ≥7.8 mmol/L and <11.1 mmol/L) or both. They can also take part in the trial if their HbA1c levels lie between 5.7 and 6.4% or they obtain a score of 12 or more in the Australian type 2 diabetes risk (AUSDRISK) tool assessment (a noninvasive questionnaire to determine the risk of developing diabetes). The participants should not take part in any clinical trial (particularly with new investigational drugs) before starting this trial. They must be readily available for 3 months and should be willing to maintain similar dietary patterns and physical activity during the trial period.

### Exclusion criteria

Participants are excluded if they are unwilling to provide blood samples during the initial and final visits. Based on the medical questionnaire they will also be excluded if they have established T2D, gall bladder problems, pacemaker implants, severe neurological diseases or seizures. Women who are pregnant or lactating will also be excluded. People who consume more than two servings of oily fish per week or take any other dietary supplements known to influence blood glucose levels are also ineligible to participate in the trial. Based on their medication intake from the medical questionnaire, participants will be excluded from the trial if they are taking aspirin, warfarin, clopidogrel, ibuprofen, naproxen, dalteparin, enoxaparin or heparin to avoid any possible drug interaction with curcumin.

### Sample size calculation

Based on the HbA1c data of the prediabetic people from other studies [27, 28], with a standard deviation of 0.5 units in HbA1c, a sample size of 17 participants in each treatment group will give 80% power to detect a 0.5-unit fall in HbA1c at a type 1 error (alpha) of 0.05. To allow for dropouts we will recruit 4 × 20 = 80 participants according to the inclusion criteria.

### Participant recruitment

Participants will be recruited from the Hunter region in NSW using media advertising and social media (Facebook and Twitter) approved by the University of Newcastle Media and Marketing Department. Recruitment flyers will be placed on notice boards at the University of Newcastle, local pharmacies, local pathology centres (with permission) and in newsletters. Participants will also be recruited from the Hunter Medical Research Institute (HMRI) Volunteer Register, as well as from clinics across John Hunter/Newcastle Community Health Centre and Belmont Hospital diabetes clinics, Medicare local and GP practice databases with consent from participating GPs. Interested participants will contact the study coordinator through email or telephone, who will screen the participants according to inclusion and exclusion criteria. Potential participants will receive a Participant Information Sheet and the Health, Diet (3-day food record) and Physical Activity Questionnaire (IPAQ long form) after telephone screening. Participants will return the completed questionnaires along with a signed Consent Form. Study investigators will use demographic and lifestyle details to determine participant suitability before visiting the NRG or JHH for a baseline visit.

### Baseline assessments

After returning their signed Consent Forms, participants are provided with baseline visit details (Table 1). They will need to complete a 3-day food record and physical activity (IPAQ) and medical questionnaires before visiting trial site. Participants will visit either NRG or JHH after a minimum of a 10-h overnight fasting. Baseline assessments include anthropometry measurements using bioelectric impedance and a 20-mL blood sample collection for assessment of plasma glucose levels, insulin, fructosamine, HbA1c, TC, TG, HDL-C, LDL-C, CRP, whole blood cell count (WBC), leptin, adiponectin and other inflammatory cytokines (IL-1β, IL-6).

**Table 1:** Study timeline and trial assessments

Assessments				
Screening (telephone)	X			
Informed consent (mail)	X			
IPAQ questionnaire (mail)	X			
3-day food record (mail)	X			
Medical questionnaire (mail)	X			
		Week 1	Week 6	Week 12
		Baseline (visit1)	Test (visit 2)	Test (visit 3)
Randomisation		X		
Intervention allocation		X		
Anthropometry measurements		X	X	X
Blood sample		X		X
Food diary		X	X	X
Physical activity		X	X	X
Biochemical analysis		X		X
Fasting plasma glucose		X		X
Fasting plasma insulin		X		X
HbA1c		X		X
Fructosamine		X		X
HOMA IR, HOMA S, HOMA β, QUICKI		X		X
Total cholesterol		X		X
Triglycerides		X		X
HDL-C, LDL-C, Total/HDL-C ratio		X		X
CRP		X		X
Whole blood cell count		X		X
Il-6, Il-1β, adiponectin, leptin		X		X
Capsule count and log record			X	X
Plasma curcumin concentration				X
Erythrocyte fatty acids content		X		X
Adverse event reporting			X	X

*IPAQ* International Physical Activity Questionnaire, *HbA1c* glycosylated haemoglobin, *HOMA IR* Homeostatic Model Assessment for Insulin Resistance, *HOMA S* Insulin sensitivity, *HOMA β* beta-cell function, *QUICKI* Quantitative insulin Sensitivity Check Index, *HDL-C* high-density lipoprotein cholesterol, *LDL-C* low-density lipoprotein cholesterol, *CRP* C-reactive protein, *IL-6* interleukin-6, *IL-1β* interleukin-1 beta

### Randomisation

Eligible participants who attend the baseline visit are randomised into treatments with either double placebo, curcumin alone, fish oil alone or double active (curcumin and fish oil) groups according to a computer-generated randomisation sequence. This randomisation is done in equal block sizes of 8 to ensure the balance between equal numbers of men and women. Randomisation is carried out by an individual who is not involved in trial data collection and analysis. Investigational product will be carefully stored (under the responsibility of the investigator) in a locked, limited-access area, safe and separate from drugs. Only authorised persons will have access to the storage place and capsules will be stored in sealed HD-PE bottles at room temperature (15–25 °C) in a dry place, protected from light and away from heat sources. The active and placebo capsules are identical, encapsulated and matched for colour. To increase the compliance of participants to the intervention, they are provided with a capsule log sheet during the study period to record their capsule intake. At the end of the study they are advised to return the capsule containers along with their log sheet.

### Intervention

Following randomisation, participants will receive one of the following interventions:

1. Placebo: 2 × 500-mg CN-Placebo capsules plus 2 × 1000-mg corn oil capsules per day
2. Curcumin (Meriva curcumin): 2 × 500-mg CN capsules (providing 180 mg curcumin/day) plus 2 × 1000-mg corn oil capsules per day
3. LCn-3PUFA (EPAX1050): 2 × 1000-mg fish oil (FO) capsules (providing 1.2 g eicosapentaenoic acid (EPA)/DHA) plus 2 × 500-mg CN-Placebo capsules per day
4. Combination (curcumin and LCn-3PUFA): 2 × 500-mg CN capsules (providing 180 mg curcumin/day) plus 2 × 1000-mg FO capsules per day (providing 1.2 g EPA/DHA)

The participants are advised to take four capsules daily (two with their morning meal and two with their evening meal) with no changes in their dietary intake or physical activity during the study period.

### Interim visit

Participants are scheduled for the interim visit after 6 weeks of the intervention period (Table 1). They will need to provide completed diet and physical activity questionnaires. Compliance for the intervention is measured by capsule count and capsule log sheet. The follow-up visit includes only a 10-h overnight fast and anthropometric assessments using bioelectric impedance. Any adverse events or reports of difficulties in compliance with the intervention are recorded during this visit.

### Post-intervention visit

The post-intervention visit is scheduled on the last day of the participant’s intervention period (Table 1). All baseline measures, including a 10-h overnight fast, anthropometric measurements and blood sample collection will be repeated on this visit. They will also be asked to fill out 3-day food record and physical activity questionnaires. Participants are advised to return capsule containers along with the capsule log sheet on the final visit day.

### Safety and compliance monitoring

During the follow-up and post-intervention visits, any serious adverse events (SAE), adverse events (AE) related to intervention, compliance with intervention, or issues with other procedures (e.g. blood collection, anthropometric measurements, etc.) are discussed with participants and recorded. The study coordinator will inform the principal investigator of all AEs and SAEs, who will then follow procedures for unblinding as necessary and notify the relevant bodies. Depending on the nature of AE or SAE, it may be necessary for treatment to cease and/or for the participant to be withdrawn from the study. Should the need for unblinding in the curcumin or LCn-3PUFA group versus placebo group arise, subjects may inform the study coordinator or anyone from the research team.

For emergency situations, subjects will be given an emergency contact card, which will provide general study information and contact information for the unbiased randomisers. Compliance of the participant to the intervention is measured using capsule count and capsule log record. Erythrocyte fatty acid analysis is used to measure participant compliance with fish oil. Serum levels of curcumin are measured using HPLC; this will be used as a measure to determine participant compliance with curcumin tablets.

### Ethics

This trial has been approved by the University of Newcastle and the Hunter New England Area Health Service Human Research Ethics Committees, Approval Nos. H-2014-0385 and HNE HREC 16/03/16/3.02, respectively. The trial is registered with the Australia New Zealand Clinical Trial Registry (Trial ID ACTRN12615000559516).

### Data collection and outcome measures

#### Primary outcome

The primary outcome measure in this trial is the difference in HbA1c levels in 12 weeks between double placebo, curcumin, LCn-3PUFA and double active groups from baseline to the post-intervention visit (Additional file 2). HbA1c is measured in blood samples obtained at baseline and post-intervention visits through the cation exchange HPLC method by Hunter New England Area Health Pathology Services (HNEHAPS) (accredited pathology laboratory for compliance with National Pathology Accreditation Advisory Council Standards).

#### Secondary outcomes

Secondary outcomes in this trial include glycaemic profiles and insulin sensitivity, Lipid profile and inflammation-related parameters (Additional file 2).

##### Glycaemic profiles and insulin sensitivity

In addition to primary outcome, we will also measure fasting glucose and insulin levels in serum samples obtained from the trial participants to evaluate the effects of curcumin and/or LCn-3PUFA on glycaemic control and insulin resistance between the four groups. These parameters will be measured by HNEHAPS. Insulin resistance will be calculated using the Homeostatic Model Assessment equations, HOMA IR (fasting insulin (U/L) × fasting glucose (mmol/L)/22.5). The beta-cell function will be measured using HOMA β (20 × fasting insulin (IU/ml)/fasting glucose (mmol/ml) − 3.5).

##### Lipid profile

Lipid profile parameters to be measured in this trial include TG, TC, LDL-C and HDL-C, and TC:HDL-C ratio. These parameters will be measured in blood samples obtained from participants at baseline and post-intervention visits by HNEHAPS.

##### Inflammatory parameters

Inflammation-related parameters, such as IL-6, IL-1β, adiponectin and leptin in blood samples of participants at baseline and post-intervention visits, will be measured using human antibody-specific Enzyme-Linked Immunosorbent Assay (ELISA) kits (R&D Systems) at our laboratory in NRG; while the other two parameters, CRP and whole blood cell count, will be analysed by HNEHAPS.

### Additional data

#### Body composition

Body weight, BMI, body fat mass, percent body fat, waist-hip ratio and skeletal muscle mass are measured using bioelectric impedance (InBody 230). These parameters are measured in a fasting state at baseline, follow-up and post-intervention visits.

#### Questionnaires

Changes in the dietary pattern, such as an increase in turmeric or fatty fish intake during the trial period, might alter the effects of intervention on clinical outcomes. To avoid this, the participants are advised to maintain a consistent dietary pattern during the trial period. They will be asked to complete a 3-day food record during each visit to enable us to get an appropriate picture of the dietary intake pattern of participants and identify any major changes in the dietary habits of participants. These food diaries will be analysed with Food Works Professional 8 2015 (Xyris software (Australia) Pvt. Ltd.). Along with dietary intake, participants will be advised to maintain similar physical activity during the 12-week trial period. The IPAQ long version is used to assess the levels of physical activity. This version allows the participants to record the number of hours or minutes that they spent on job-related physical activity, travel-related physical activity and recreation- or sport-related physical activity during the last 7 days. It also has a provision for recording the number of days, hours or minutes participant spend on walking and sitting. The physical activity of the participant will be reported in metabolic equivalents (METs)-minutes/week. This will also help us to determine the impact of different levels of physical activity on intervention.

### Data analysis

Data obtained from the all participants will be analysed according to the intention-to-treat principle. In case of missing data or dropouts, pairwise deletion will be used to analyse the significant effect of intervention in participants who have completed the trial. Normality of baseline data will be examined using histograms with a normal distribution curve overlayed and the Shapiro-Wilk test. Based on the distribution of data, the outcome measures will be analysed using analysis of variance (ANOVA) (normal distribution) or the Wilcoxon signed rank test (nonparametric data). Two-way ANOVA with post hoc comparisons (Bonferroni correction) will be used to determine the effect of intervention on different variables and also to determine synergistic and/or complimentary effects between the two interventions (curcumin and LCn-3PUFA). ANCOVA will be used to assess the effects of confounding factors on treatment that include age, gender, BMI, physical activity levels and dietary intake. Significance will be set at a *P* value < 0.05. Statistical analysis will be performed using GraphPad Prism version 6 and IBM SPSS 22 software.

## Discussion

Parallel to an increase in the number of individuals with T2D, the prevalence of people with prediabetes is also increasing exponentially [5]. Currently, prediabetes is managed by lifestyle modifications to delay its onset or prevent its progression to T2D. To date there are no pharmacological agents approved for the treatment of prediabetes. Lifestyle modifications alone are insufficient to bring down the prevalence of prediabetes, so there is a need for long-term, safe and cost-effective agents that can act through multiple mechanisms to improve insulin sensitivity and beta-cell function. In this trial we are evaluating the effects of the diet-derived bioactives curcumin and/or LCn-3PUFA. These are derived from turmeric powder and seafood, respectively, and have the potential to influence multiple mechanisms. To the best of our knowledge, to date this is the first clinical trial designed to evaluate the combination of curcumin and LCn-3PUFA in prediabetes. If the application of curcumin and/or LCn-3PUFA is successful in improving insulin sensitivity and glycaemic profiles in this trial, this combination could be examined in large-scale trials that could provide a better alternative for the management of prediabetes to prevent or delay the onset of T2D. In addition to the effects of curcumin and LCn-3PUFA on glycaemic profiles, this combination might also improve underlying chronic low-grade inflammation, which is involved in down-regulation of insulin signalling and activity pathways. The anti-inflammatory and triglyceride-lowering effects of curcumin and LCn-3PUFA might also provide an alternative option to reduce cardiovascular risk factors in those with prediabetes, which is not seen with conventional oral antidiabetic agents. Therefore, this trial might be an important step in evaluating the effects of bioactives for alleviating both hyperglycaemic and cardiovascular risk factors in individuals who are at high risk of developing T2D.

Curcumin, an anti-inflammatory bioactive derived from the spice turmeric has, in preclinical and in-vitro studies, been shown to act on multiple targets for delaying the risk of developing T2D; via down-regulation of low-grade inflammation, cytoprotection of beta-cells, improvement in beta-cell function and decreasing fat accumulation in nonadipose tissues. Curcumin has been shown to decrease inflammation in adipocytes via multiple pathways such as inhibition of macrophage infiltration, scavenging ROS and down-regulation of proinflammatory kinase (NFκB and JKK) signalling. In-vivo studies with curcumin supplementation in high-fat-diet-fed genetically obese (ob/ob) mice resulted in amelioration of diabetes-associated symptoms along with a decrease in insulin resistance [29]. Multiple mechanisms, like decreased macrophage accumulation in WAT, down-regulation of proinflammatory cytokines and increase in adiponectin levels, are exhibited by curcumin for improving insulin sensitivity [29]. Curcumin increased the expression of AMPK in diabetic db/db mice, which might be a possible mechanism in suppressing hepatic glucose production, resulting in decreased blood glucose levels [30]. Curcumin exhibits cytoprotective mechanisms increasing levels of key mitochondrial biogenesis-regulating transcription factors like PGC-1α, nuclear respiratory factor-1 and mitochondrial transcription factor A [31]. Stimulation of volume-regulated anion channels may be one of the primary mechanisms for the antihyperglycaemic effects of curcumin. Curcumin directly stimulated the volume-regulated anion channels in rat pancreatic beta-cells. This effect was accompanied by generation of electrical activity and depolarisation which resulted in enhanced insulin release [32]. Curcuminoids increased the expression of the antioxidant enzymes heme-oxygenase 1 and NADPH:quinone oxidoreductase 1 at messenger ribonucleic acid (mRNA) levels up to 12-fold and at protein levels by 6-fold in human islets (used for human transplantation) [33]. These observations suggest that curcumin might be beneficial in improving cellular defense mechanisms against oxidative stress. Direct stimulation of pancreatic beta-cells and cellular defence against oxidative stress are two mechanisms which provide considerable evidence for exploring the potential of curcumin in controlling or preventing T2D.

Meta-analyses and randomised controlled trials have reported that LCn-3PUFA have either no effects or worsening effects on glycaemic profiles and insulin sensitivity [34, 35]. On the contrary, epidemiological studies have reported an inverse correlation between regular consumption of fish and prevalence of diabetes [36–40]. A recently published prospective study of a cohort of 407 obese and IGT participants demonstrated that elevated plasma levels of LCn-3PUFA are associated with a lower prevalence of T2D over the extended 11-year follow-up period [41]. Elevated serum EPA, DHA and DPA levels are associated with higher insulin sensitivity, providing a plausible explanation for the lower prevalence rates of diabetes [41]. In line with epidemiological studies, in-vitro and preclinical studies have reported beneficial effects of LCn-3PUFA on glycaemic profiles and insulin resistance through amelioration of chronic low-grade inflammation and lipotoxicity. Administration of LCn-3PUFA in the diet (5–44%) protected the high-fat-diet-fed animals from developing lipid abnormalities and impaired glucose homeostasis [42–44]. LCn-3PUFA are natural agonists of PPARα subunits; the carboxylic group and the hydrophobic chain in the structure of LCn-3PUFA allows optimal binding to PPARα [45]. Activation of PPARα by LCn-3PUFA results in increased fatty acid β-oxidation in peroxisomes (to a lesser extent in mitochondria) and decreased lipogenesis. The increase in fatty acid oxidation leads to decreased accumulation of fatty acids (lipotoxicity) in the liver along with improvement in insulin sensitivity in hepatocytes [45]. Stimulation of AMPK by LCn-3PUFA, along with its effects on PPARα and SREBP-1c, results in significant reduction in triglycerides with overall improvement in insulin sensitivity of hepatocytes. LCn-3PUFA, through PPARγ stimulation, has a mechanism of action similar to that of the thiazolidinedione class of antidiabetic drugs [46], balances adipokine secretion, decreases proinflammatory signalling and increases insulin sensitivity [47–49]. LCn-3PUFA has been shown in in-vitro studies to decrease diacylglycerol, muscle ceramide and long-chain acyl CoA accumulation in myotubes that protect high-fat-diet-fed rodents from developing insulin resistance and improved glycogen synthesis and glucose uptake in skeletal muscle [50–53]. Clinical studies with supplementation of EPA + DHA at dose of 3.1–8.4 g/day have reported a 30–55% decrease in the production of ROS by cultured human neutrophils [54, 55]. Cell culture studies have reported EPA and DHA to have potency to inhibit the expression of the proinflammatory cytokines IL-1β, tumour necrosis factor alpha (TNFα) and NFκB by macrophages [56–58]. Recent human intervention studies with LCn-3PUFA at dose levels between 2 and 4 g/day reported a decrease in inflammation and proinflammatory markers [59, 60]. A recently published research study on G-protein-coupled receptors (GPR) reported that the anti-inflammatory activity of LCn-3PUFA is mediated through the activation of GPR120 [61]. LCn-3PUFA stimulates GPR120 in macrophages and adipocytes, through which it exhibits its anti-inflammatory mechanisms. This involves inhibition of transforming growth factor beta (TGF-beta)-activated kinase 1 (TAK 1) through a β-arrestin1/TGF-beta-activated kinase 1/MAP3K7 binding protein 1 (TAB1)-dependent mechanism. This inhibition leads to down-regulation in proinflammatory pathways like IKK/NFκB and JNK/AP1 [61]. LCn-3PUFA abolishes activation of the NLRP3 inflammasome along with inhibition of caspase-1 activation and IL-1β secretion [62]. The established data on anti-inflammatory (inhibition of inflammasome and GPR120 activation) and triglyceride-reducing effects of LCn-3PUFA might be an important aspect to be considered for reducing low-grade systemic inflammation, which is a promoter for several pathological mechanisms, like glucotoxicity and lipotoxicity, in different tissues. Regardless of the availability of large-volume, ambiguous clinical data, cellular and physiological mechanisms in different tissues still make LCn-3PUFA look like a promising agent for delaying or reducing the risk of T2D.

The combination of curcumin and LCn-3PUFA might provide either synergistic or complimentary effects in prediabetes, which is evaluated through this 2 × 2 factorial study design. To date previous studies have not reported curcumin interfering with LCn-3PUFA binding to GPR120, thereby preventing the competitive binding for the same receptor. Substantiating evidence of the blood-glucose-lowering effects of curcumin and the triglyceride-lowering effects of LCn-3PUFA might produce improved metabolic health in prediabetics. The cytoprotective effects of curcumin might also provide additional benefits to maintain the integrity of pancreatic beta-cells. This trial, therefore, looks at the potential of these two bioactives in decreasing the risk factors for developing T2D.

### Strengths and limitation of study design

The strengths of this COP-D trial include valid study methodology; factorial design to evaluate the individual effect and complimentary or synergistic effects of both interventions; and double blinding of both participants and investigators to reduce bias. The participants are advised to maintain a constant dietary and physical activity pattern, which is evaluated though questionnaires, to reduce the effect of confounding factors on intervention. The use of a capsule log, a capsule count, erythrocyte fatty acid estimation using gas chromatography and measurement of serum curcumin levels using HPLC provides the best measure of compliance with the interventions. No toxic or adverse events in previous studies have been reported at the dose of curcumin and LCn-3PUFAs used in this trial. There might also be potential limitations for this study because as we are measuring only risk factors like fasting glucose and insulin, lipid parameters and proinflammatory cytokines, we cannot translate these results completely into preventing or delaying the onset T2D. A follow-up study with a larger sample size and prolonged duration would be required to substantiate whether curcumin and/or LCn-3PUFA could actually prevent or delay the onset of T2D. If we can obtain considerable evidence of the beneficial effects of curcumin and LCn-3PUFA this trial can provide the basis for a safe and cost-effective approach to decrease the risk of T2D.

### Trial status

Recruitment for this trial started in July 2015. This study is currently ongoing and is actively recruiting participants from local communities of NSW, Australia. No changes have been made to the clinical protocol post recruitment of participants. To date 38 participants completed all trial visits. It is expected to take approximately 2 years (from the starting day of trial recruitment) to complete the study.

## Additional files

Additional file 1: The SPIRIT Checklist. (DOC 123 kb)
Additional file 2: Outcome measures. (PPTX 46 kb)

## Abbreviations

AMPK: 5' AMP-activated protein kinase
AUSDRISK: Australian type 2 diabetes risk assessment tool
CRP: C-reactive protein
HbA1c: Glycosylated haemoglobin
HDL-C: High-density lipoprotein cholesterol
HOMA: Homeostatic Model Assessment
IKK: Inhibitor of kappa B kinase
IL: Interleukin
IPAQ: International Physical Activity Questionnaire
JNK: c-Jun N-terminal kinases
LCn-3PUFA: Long-chain omega-3 polyunsaturated fatty acids
LDL: Low-density lipoprotein
NFκB: Nuclear factor kappa B
OGTT: Oral glucose tolerance test
PPARγ: Peroxisome proliferator-activated receptor gamma
QUICKI: Quantitative Insulin Sensitivity Index
TC: Total cholesterol
TGA: Therapeutic Goods Administration
