# Supplementary material for: Curcumin and long-chain Omega-3 polyunsaturated fatty acids for Prevention of type 2 Diabetes (COP-D): study protocol for a randomised controlled trial
Source: Trials. 2016 Nov 29;17:565. doi: 10.1186/s13063-016-1702-9 (PMC5126844; doi:10.1186/s13063-016-1702-9)
Supplement: Additional file 2: — Outcome measures. (PPTX 46 kb) [file 13063_2016_1702_MOESM2_ESM.pptx]

## Slide 1
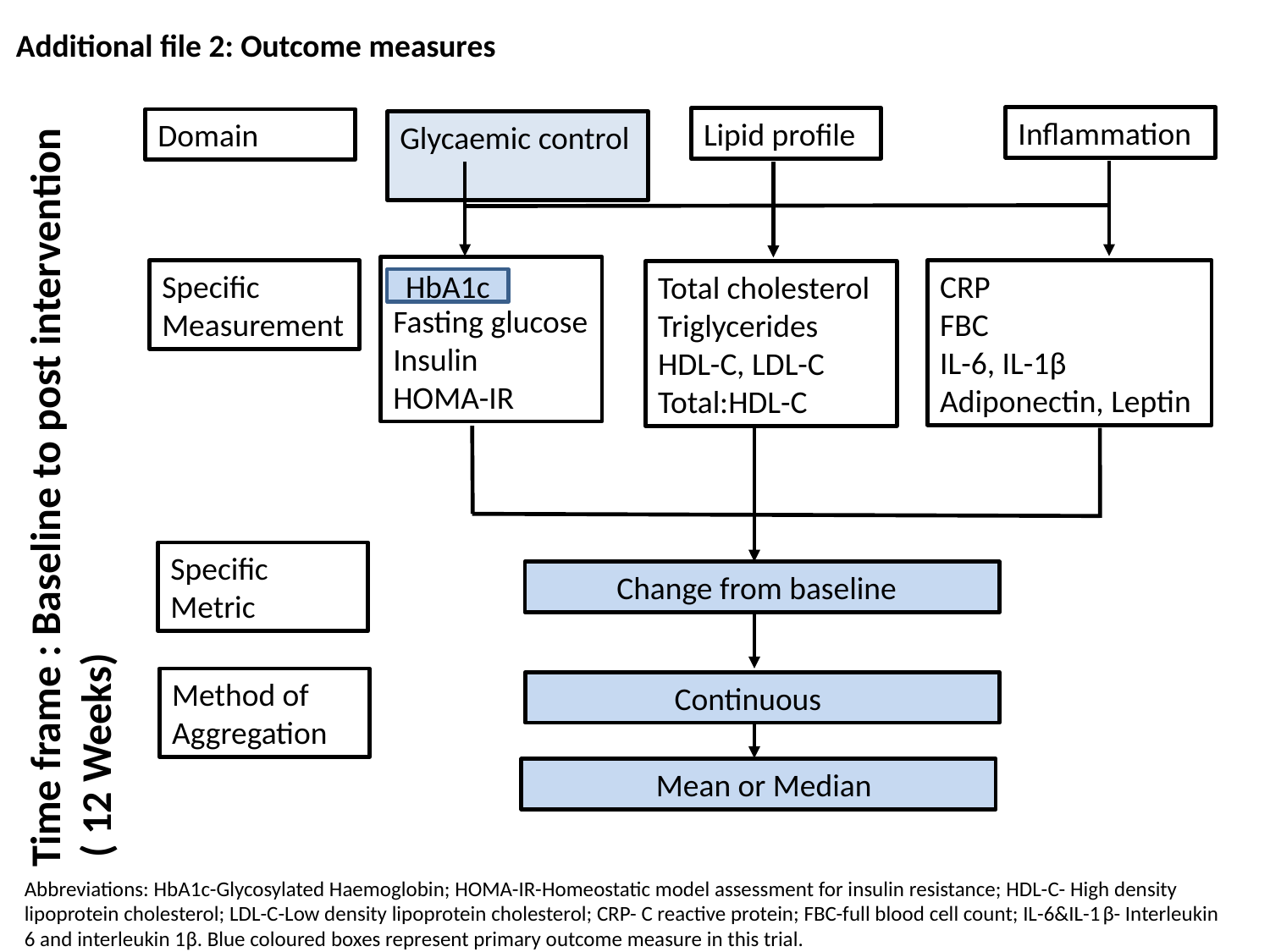

Additional file 2: Outcome measures
Inflammation
Lipid profile
Domain
Glycaemic control
HbA1c
Fasting glucose
Insulin
HOMA-IR
CRP
FBC
IL-6, IL-1β
Adiponectin, Leptin
Specific Measurement
Total cholesterol
Triglycerides
HDL-C, LDL-C
Total:HDL-C
HbA1c
Time frame : Baseline to post intervention
 ( 12 Weeks)
Specific Metric
 Change from baseline
Method of Aggregation
 Continuous
 Mean or Median
# Abbreviations: HbA1c-Glycosylated Haemoglobin; HOMA-IR-Homeostatic model assessment for insulin resistance; HDL-C- High density lipoprotein cholesterol; LDL-C-Low density lipoprotein cholesterol; CRP- C reactive protein; FBC-full blood cell count; IL-6&IL-1β- Interleukin 6 and interleukin 1β. Blue coloured boxes represent primary outcome measure in this trial.
